# Special issue on nanofluids

**DOI:** 10.1186/1556-276X-6-99

**Published:** 2011-01-25

**Authors:** Stephen US Choi, Yogesh Jaluria, Oronzio Manca, Liqiu Wang

**Affiliations:** 1 Department of Mechanical and Industrial Engineering, University of Illinois at Chicago, Chicago, IL 60607 USA; 2 Department of Mechanical and Aerospace Engineering, Rutgers, The State University of New Jersey, Piscataway, NJ 08854-8058, USA; 3 Dipartimento di Ingegneria Aerospaziale e Meccanica, Seconda Universita' degli Studi di Napoli Real Casa dell'Annunziata, Via Roma 29, Aversa (CE) 81031, Italy; 4 Department of Mechanical Engineering, The University of Hong Kong, Pokfulam Road, Hong Kong

## Editorial

Nanofluids, or fluid suspensions of nanometer-sized structures, are research challenges of rare potential but daunting difficulty. The potential comes from both scientific and practical opportunities in many fields. The difficulty reflects the issues related to multiscales. Nanofluids involve at least four relevant scales: the molecular scale, the microscale, the macroscale, and the systemscale. The molecular scale is characterized by the mean free path between molecular collisions, the microscale by the smallest scale at which the law of continuum mechanics applies, the macroscale by the smallest scale at which a set of averaged properties of concern can be defined, and the systemscale by the length scale corresponding to the domain of interest. By their very nature, research and engineering practice in nanofluids are aimed at enhancing fluid macroscale and system-scale properties through the manipulatationof microscale physics (structures, properties and activities). Therefore, the success of nanofluid technology depends very much on how well we can address issues such as effective means of microscale manipulation, interplay among physics at different scales, and the optimization of microscale physics for the optimal macroscale and system-scale properties.

The present special issue is dedicated to the latest advances in addressing these issues. The objective is to promote interdisciplinary research on nanofluids and motivate the nano community, which is uniquely qualified to make valuable contributions, to become more involved in this field of research and development.

We would like to express our sincere appreciation to the contributing authors and reviewers who have maintained the standard associated with *Nanoscale Research Letters*. We are also thankful to Professor Zhiming Wang, the Editor-in-Chief, for providing this forum to discuss this emerging field.

